# Ethyl 4-cyano-7-nitro-1,2,3,3a,4,5-hexa­hydro­pyrrolo­[1,2-*a*]quinoline-4-carboxyl­ate

**DOI:** 10.1107/S1600536812003480

**Published:** 2012-01-31

**Authors:** Yvon Bibila Mayaya Bisseyou, Adéyolé Timotou, Ajouby Adjou, Rita Kakou-Yao, Jules Tenon Abodou

**Affiliations:** a Laboratoire de Cristallographie et Physique Moléculaire, UFR SSMT, Université de Cocody, 22 BP 582 Abidjan 22, Cote d’Ivoire; b Laboratoire de Chimie Organique Structurale, UFR SSMT, Université de Cocody, 22 BP 582 Abidjan 22, Cote d’Ivoire

## Abstract

In the title compound, C_16_H_17_N_3_O_4_, the six-membered N-containing ring adopts a half-chair conformation. One C atom of the five-membered ring is disordered over two sites, with occupancy factors of *ca* 0.67 and 0.33. The major pyrroline component adopts a half-chair conformation. Inter­molecular C—H⋯O hydrogen bonds forming centrosymmetric dimers are observed in the crystal.

## Related literature

For the biological activity of tricyclic quinoline derivatives, see: Dalla Via *et al.* (2008 ▶); Gasparotto *et al.* (2006 ▶); Ferlin *et al.* (2000 ▶). For the crystal structure of an inter­mediate compound, see: Yapo, Konan *et al.* (2010 ▶). For a closely related crystal structure, see: Yapo, Abou *et al.* (2010 ▶). For ring conformation analysis, see: Cremer & Pople (1975 ▶). For graph-set notation, see: Bernstein *et al.* (1995 ▶).
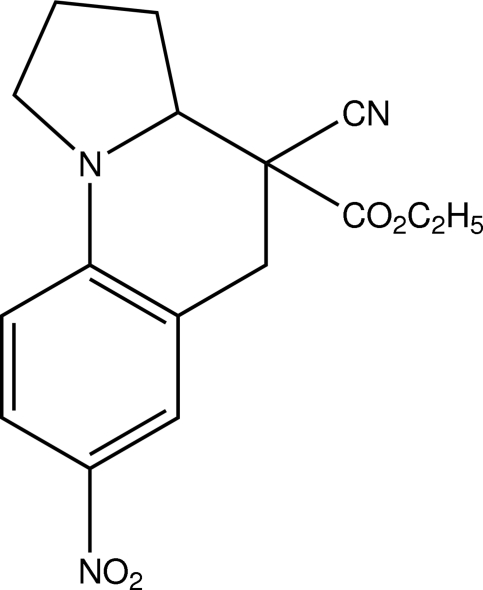


## Experimental

### 

#### Crystal data

C_16_H_17_N_3_O_4_
*M* *_r_* = 315.33
Triclinic, 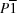

*a* = 7.2292 (2) Å
*b* = 9.1589 (3) Å
*c* = 11.8243 (5) Å
α = 79.332 (1)°
β = 82.609 (1)°
γ = 80.429 (2)°
*V* = 754.79 (5) Å^3^
*Z* = 2
Mo *K*α radiation
μ = 0.10 mm^−1^
*T* = 223 K
0.25 × 0.20 × 0.15 mm

#### Data collection

Nonius KappaCCD area-detector diffractometer
9677 measured reflections
3879 independent reflections
2498 reflections with *I* > 2σ(*I*)
*R*_int_ = 0.049

#### Refinement

*R*[*F* ^2^ > 2σ(*F* ^2^)] = 0.083
*wR*(*F* ^2^) = 0.247
*S* = 1.17
3879 reflections
214 parameters
12 restraints
H-atom parameters constrained
Δρ_max_ = 0.75 e Å^−3^
Δρ_min_ = −0.61 e Å^−3^

### 

Data collection: *COLLECT* (Nonius, 2001 ▶); cell refinement: *DENZO* and *SCALEPACK* (Otwinowski & Minor, 1997 ▶); data reduction: *DENZO* and *SCALEPACK*; program(s) used to solve structure: *SHELXS97* (Sheldrick, 2008 ▶); program(s) used to refine structure: *SHELXL97* (Sheldrick, 2008 ▶); molecular graphics: *ORTEP-3 for Windows* (Farrugia, 1997 ▶) and *PLATON* (Spek, 2009 ▶); software used to prepare material for publication: *WinGX* (Farrugia, 1999 ▶).

## Supplementary Material

Crystal structure: contains datablock(s) I, global. DOI: 10.1107/S1600536812003480/wn2466sup1.cif

Structure factors: contains datablock(s) I. DOI: 10.1107/S1600536812003480/wn2466Isup2.hkl

Supplementary material file. DOI: 10.1107/S1600536812003480/wn2466Isup3.cml

Additional supplementary materials:  crystallographic information; 3D view; checkCIF report

## Figures and Tables

**Table 1:** Hydrogen-bond geometry (Å, °)

*D*—H⋯*A*	*D*—H	H⋯*A*	*D*⋯*A*	*D*—H⋯*A*
C11—H11*A*⋯O3^i^	0.97	2.48	3.432 (3)	167

Symmetry code: (i) 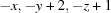.

**Fig. 1.**
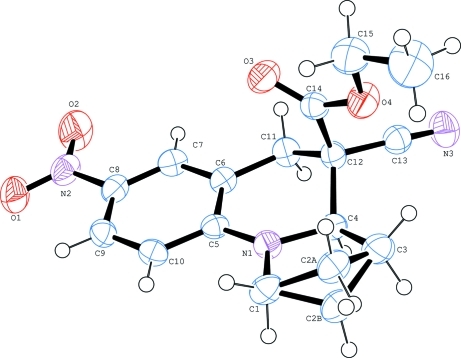
ORTEP view of the molecular structure of the title compound. Displacement ellipsoids are drawn at the 50% probability level and H atoms are shown as small spheres of arbitrary radius. C2A and C2B are the major and minor components, respectively, of the disordered atom.

**Fig. 2.**
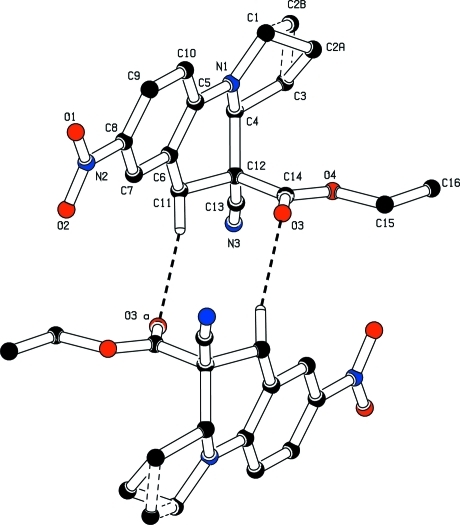
Part of the crystal packing showing a centrosymmetric R22(10) dimer unit. For the sake of clarity, the unit-cell outline and H atoms not involved in hydrogen bonds have been omitted. Dashed lines indicate hydrogen bonds. Atom O3a belongs to the molecule at symmetry position (-x+2,-y,-z+1).

**Fig. 3.**
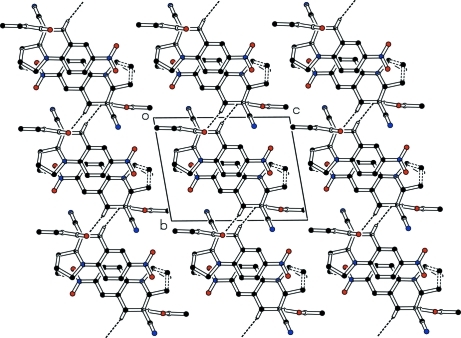
Packing diagram of the title compound, viewed down the a axis. H atoms not involved in hydrogen bonds have been omitted for clarity. Dashed lines indicate hydrogen bonds.

## supplementary crystallographic information

### Comment

The title compound is a tricyclic quinoline derivative obtained from an intermediate compound, (*E*)-ethyl 2-cyano-3-[5-nitro-2-(pyrrolidin-1-yl)phenyl]acrylate, whose molecular and crystal structures were recently determined by X-ray diffraction (Yapo, Konan *et al.*, 2010). Tricyclic quinoline derivatives have received considerable attention because of their important therapeutic properties (Dalla Via *et al.*, 2008; Gasparotto *et al.*, 2006; Ferlin *et al.*, 2000).

In this paper the crystal structure of the title compound is reported from single-crystal X-ray diffraction data collected at 223 K. The molecular structure of the title compound is shown in Fig. 1. The structure is composed of two principal parts: the quinoline ring system and the pyrroline ring.

The quinoline ring system has geometrical parameters which are consistent with those reported recently (Yapo, Abou *et al.*, 2010). The six-membered N-containing ring adopts a half-chair conformation, with puckering parameters Q = 0.512 (2)Å, θ = 129.6 (2)°, φ = 283.6 (3)° (Cremer & Pople, 1975). The pyrroline ring exhibits disorder of atom C2 over two sites, with occupancy factors of 0.672 (5) and 0.328 (5). The major component of the five-membered ring adopts a half-chair conformation with puckering parameters Q(2) = 0.335 (3) Å and φ = 54.5 (5)° .

In the crystal structure, molecules form centrosymmetric dimeric units *via* C—H···O hydrogen bonds, characterized by an *R*^2^_2_(10) (Bernstein *et al.*, 1995) motif (Fig. 2). These centrosymmetric *R*^2^_2_(10) dimers are arranged in the crystal structure as shown in Fig. 3.

### Experimental

(*E*)-Ethyl-2-cyano-3-(5-nitro-2-pyrrolidin-1-yl)phenyl) acrylate (2 g, 6.34 mmol) was dissolved in anhydrous dimethylformamide (10 ml). The mixture was heated to reflux over a period of 24 h. After cooling to ambient temperature, the reaction mixture was poured into water (20 ml). After extraction by ethyl acetate (2x50ml), the organic layers were dried over magnesium sulfate, filtered and concentrated under reduced pressure. The residue was purified by chromatography on silica gel. Elution solvent: hexane/ethyl acetate (90/10). Yellow single crystals of the title compound were obtained with a yield of 48% (m.p.: 397–398 K; Rf: 0.65, hexane/ethyl acetate: 90/10).

### Refinement

H atoms were placed in idealized positions and allowed to ride on their parent atoms, with Csp^2^—H = 0.93 Å, C(methine)—H = 0.98 Å, C(methylene)—H = 0.97 Å, C(methyl)—H = 0.96 Å; *U*_iso_(H) = x*U*_eq_(C), where x = 1.5 for methyl H and 1.2 for all other H atoms.

### Crystal data

**Table:**

C_16_H_17_N_3_O_4_	*Z* = 2
*M**_r_* = 315.33	*F*(000) = 332
Triclinic, *P*1	*D*_x_ = 1.387 Mg m^−^^3^
*a* = 7.2292 (2) Å	Mo *K*α radiation, λ = 0.71073 Å
*b* = 9.1589 (3) Å	Cell parameters from 9677 reflections
*c* = 11.8243 (5) Å	θ = 1.8–29.2°
α = 79.332 (1)°	µ = 0.10 mm^−^^1^
β = 82.609 (1)°	*T* = 223 K
γ = 80.429 (2)°	Prism, yellow
*V* = 754.79 (5) Å^3^	0.25 × 0.20 × 0.15 mm

### Data collection

**Table:**

Nonius KappaCCD area-detector diffractometer	2498 reflections with *I* > 2σ(*I*)
Radiation source: fine-focus sealed tube	*R*_int_ = 0.049
graphite	θ_max_ = 29.2°, θ_min_ = 1.8°
φ and ω scans	*h* = 0→9
9677 measured reflections	*k* = −11→12
3879 independent reflections	*l* = −15→16

### Refinement

**Table:**

Refinement on *F*^2^	Secondary atom site location: difference Fourier map
Least-squares matrix: full	Hydrogen site location: inferred from neighbouring sites
*R*[*F*^2^ > 2σ(*F*^2^)] = 0.083	H-atom parameters constrained
*wR*(*F*^2^) = 0.247	*w* = 1/[σ^2^(*F*_o_^2^) + (0.1394*P*)^2^ + 0.0744*P*] where *P* = (*F*_o_^2^ + 2*F*_c_^2^)/3
*S* = 1.17	(Δ/σ)_max_ < 0.001
3879 reflections	Δρ_max_ = 0.75 e Å^−^^3^
214 parameters	Δρ_min_ = −0.61 e Å^−^^3^
12 restraints	Extinction correction: *SHELXL97* (Sheldrick, 2008), Fc^*^=kFc[1+0.001xFc^2^λ^3^/sin(2θ)]^-1/4^
Primary atom site location: structure-invariant direct methods	Extinction coefficient: 0.38 (4)

### Special details

**Table:**

Geometry. All e.s.d.'s (except the e.s.d. in the dihedral angle between two l.s. planes) are estimated using the full covariance matrix. The cell e.s.d.'s are taken into account individually in the estimation of e.s.d.'s in distances, angles and torsion angles; correlations between e.s.d.'s in cell parameters are only used when they are defined by crystal symmetry. An approximate (isotropic) treatment of cell e.s.d.'s is used for estimating e.s.d.'s involving l.s. planes.
Refinement. Refinement of *F*^2^ against ALL reflections. The weighted *R*-factor *wR* and goodness of fit *S* are based on *F*^2^, conventional *R*-factors *R* are based on *F*, with *F* set to zero for negative *F*^2^. The threshold expression of *F*^2^ > σ(*F*^2^) is used only for calculating *R*-factors(gt) *etc*. and is not relevant to the choice of reflections for refinement. *R*-factors based on *F*^2^ are statistically about twice as large as those based on *F*, and *R*- factors based on ALL data will be even larger.

### Fractional atomic coordinates and isotropic or equivalent isotropic displacement parameters (Å^2^)

**Table:**

	*x*	*y*	*z*	*U*_iso_*/*U*_eq_	Occ. (<1)
N1	0.2977 (3)	0.58327 (19)	0.70594 (15)	0.0351 (5)	
C1	0.2805 (3)	0.4588 (3)	0.80197 (18)	0.0412 (6)	
H1A	0.3981	0.3908	0.8092	0.049*	
H1B	0.1808	0.4032	0.7947	0.049*	
C2A	0.2298 (6)	0.5486 (4)	0.9047 (3)	0.0419 (8)	0.672 (5)
H2A1	0.0953	0.5827	0.9146	0.050*	0.672 (5)
H2A2	0.2676	0.4865	0.9761	0.050*	0.672 (5)
C2B	0.3452 (12)	0.5011 (9)	0.9026 (6)	0.0419 (8)	0.328 (5)
H2B1	0.4735	0.4537	0.9134	0.050*	0.328 (5)
H2B2	0.2642	0.4719	0.9724	0.050*	0.328 (5)
C5	0.2756 (3)	0.5797 (2)	0.59352 (17)	0.0308 (5)	
C8	0.2277 (3)	0.5789 (3)	0.36579 (18)	0.0367 (5)	
O3	−0.0755 (2)	0.8596 (2)	0.64410 (15)	0.0488 (5)	
C7	0.2484 (3)	0.7115 (3)	0.39876 (18)	0.0365 (5)	
H7	0.2451	0.7994	0.3446	0.044*	
C10	0.2506 (3)	0.4473 (2)	0.55766 (19)	0.0352 (5)	
H10	0.2504	0.3591	0.6112	0.042*	
C4	0.3558 (3)	0.7113 (2)	0.74133 (18)	0.0348 (5)	
H4	0.4904	0.7105	0.7159	0.042*	
C11	0.2999 (3)	0.8593 (2)	0.54725 (18)	0.0369 (5)	
H11A	0.2216	0.9420	0.5043	0.044*	
H11B	0.4302	0.8752	0.5273	0.044*	
O4	−0.0134 (2)	0.8945 (2)	0.81674 (14)	0.0480 (5)	
O1	0.1913 (3)	0.4596 (2)	0.21612 (16)	0.0595 (6)	
C6	0.2741 (3)	0.7141 (2)	0.51212 (17)	0.0326 (5)	
N2	0.2021 (3)	0.5793 (3)	0.24618 (17)	0.0465 (5)	
O2	0.1926 (4)	0.6986 (2)	0.17861 (16)	0.0722 (7)	
C12	0.2475 (3)	0.8588 (2)	0.67809 (17)	0.0332 (5)	
C9	0.2265 (3)	0.4462 (3)	0.4446 (2)	0.0375 (5)	
H9	0.2097	0.3582	0.4212	0.045*	
C14	0.0328 (3)	0.8707 (2)	0.70870 (18)	0.0344 (5)	
N3	0.3742 (3)	1.0864 (2)	0.72921 (19)	0.0522 (6)	
C13	0.3147 (3)	0.9878 (3)	0.70977 (19)	0.0389 (5)	
C3	0.3340 (4)	0.6763 (3)	0.8734 (2)	0.0485 (6)	
H3A	0.4566	0.6512	0.9028	0.058*	
H3B	0.2651	0.7623	0.9053	0.058*	
C15	−0.2116 (4)	0.8961 (3)	0.8615 (2)	0.0541 (7)	
H15A	−0.2559	0.8064	0.8495	0.065*	
H15B	−0.2880	0.9831	0.8219	0.065*	
C16	−0.2252 (5)	0.9021 (4)	0.9866 (3)	0.0725 (9)	
H16A	−0.1379	0.8220	1.0232	0.109*	
H16B	−0.3511	0.8914	1.0207	0.109*	
H16C	−0.1953	0.9968	0.9969	0.109*	

### Atomic displacement parameters (Å^2^)

**Table:**

	*U*^11^	*U*^22^	*U*^33^	*U*^12^	*U*^13^	*U*^23^
N1	0.0446 (11)	0.0312 (9)	0.0282 (9)	−0.0060 (7)	−0.0043 (7)	−0.0011 (7)
C1	0.0448 (13)	0.0414 (12)	0.0345 (12)	−0.0088 (10)	−0.0089 (9)	0.0067 (9)
C2A	0.0446 (19)	0.0502 (19)	0.0286 (14)	−0.0076 (16)	−0.0043 (15)	0.0000 (13)
C2B	0.0446 (19)	0.0502 (19)	0.0286 (14)	−0.0076 (16)	−0.0043 (15)	0.0000 (13)
C5	0.0278 (10)	0.0339 (10)	0.0285 (10)	−0.0011 (8)	−0.0013 (8)	−0.0037 (8)
C8	0.0303 (11)	0.0500 (13)	0.0301 (11)	−0.0029 (9)	−0.0021 (8)	−0.0110 (9)
O3	0.0427 (10)	0.0580 (11)	0.0475 (10)	−0.0053 (8)	−0.0129 (8)	−0.0095 (8)
C7	0.0353 (11)	0.0414 (11)	0.0299 (11)	−0.0042 (9)	−0.0003 (8)	−0.0014 (8)
C10	0.0342 (11)	0.0329 (10)	0.0366 (11)	−0.0028 (8)	−0.0001 (9)	−0.0054 (8)
C4	0.0345 (11)	0.0350 (11)	0.0345 (11)	−0.0019 (8)	−0.0063 (8)	−0.0058 (8)
C11	0.0454 (12)	0.0344 (11)	0.0299 (11)	−0.0104 (9)	−0.0007 (9)	−0.0006 (8)
O4	0.0382 (9)	0.0667 (11)	0.0412 (9)	−0.0079 (8)	−0.0005 (7)	−0.0168 (8)
O1	0.0681 (13)	0.0687 (13)	0.0492 (11)	−0.0066 (10)	−0.0115 (9)	−0.0284 (9)
C6	0.0319 (10)	0.0356 (11)	0.0294 (10)	−0.0054 (8)	−0.0015 (8)	−0.0041 (8)
N2	0.0432 (11)	0.0633 (14)	0.0342 (10)	−0.0040 (10)	−0.0055 (8)	−0.0141 (9)
O2	0.1117 (19)	0.0696 (13)	0.0376 (11)	−0.0166 (12)	−0.0238 (11)	−0.0003 (9)
C12	0.0382 (11)	0.0316 (10)	0.0304 (10)	−0.0066 (8)	−0.0027 (8)	−0.0059 (8)
C9	0.0331 (11)	0.0392 (11)	0.0420 (12)	−0.0027 (9)	−0.0024 (9)	−0.0150 (9)
C14	0.0383 (11)	0.0293 (10)	0.0346 (11)	−0.0034 (8)	−0.0051 (9)	−0.0033 (8)
N3	0.0609 (14)	0.0443 (12)	0.0561 (14)	−0.0191 (10)	−0.0045 (10)	−0.0106 (10)
C13	0.0408 (12)	0.0392 (12)	0.0365 (12)	−0.0057 (9)	−0.0052 (9)	−0.0049 (9)
C3	0.0611 (16)	0.0466 (13)	0.0353 (12)	0.0052 (11)	−0.0143 (11)	−0.0053 (10)
C15	0.0392 (14)	0.0663 (17)	0.0566 (16)	−0.0072 (12)	0.0027 (11)	−0.0148 (13)
C16	0.0599 (19)	0.098 (2)	0.0578 (18)	−0.0145 (17)	0.0146 (14)	−0.0208 (17)

### Geometric parameters (Å, °)

**Table:**

N1—C5	1.366 (3)	C4—C3	1.528 (3)
N1—C4	1.455 (3)	C4—C12	1.556 (3)
N1—C1	1.461 (3)	C4—H4	0.9800
C1—C2B	1.470 (7)	C11—C6	1.510 (3)
C1—C2A	1.562 (4)	C11—C12	1.544 (3)
C1—H1A	0.9700	C11—H11A	0.9700
C1—H1B	0.9700	C11—H11B	0.9700
C2A—C3	1.461 (4)	O4—C14	1.327 (3)
C2A—H2A1	0.9700	O4—C15	1.461 (3)
C2A—H2A2	0.9700	O1—N2	1.231 (3)
C2B—C3	1.568 (8)	N2—O2	1.226 (3)
C2B—H2B1	0.9700	C12—C13	1.475 (3)
C2B—H2B2	0.9700	C12—C14	1.538 (3)
C5—C10	1.402 (3)	C9—H9	0.9300
C5—C6	1.415 (3)	N3—C13	1.133 (3)
C8—C7	1.379 (3)	C3—H3A	0.9700
C8—C9	1.389 (3)	C3—H3B	0.9700
C8—N2	1.449 (3)	C15—C16	1.480 (4)
O3—C14	1.190 (3)	C15—H15A	0.9700
C7—C6	1.382 (3)	C15—H15B	0.9700
C7—H7	0.9300	C16—H16A	0.9600
C10—C9	1.373 (3)	C16—H16B	0.9600
C10—H10	0.9300	C16—H16C	0.9600
			
C5—N1—C4	122.46 (16)	C6—C11—H11B	109.2
C5—N1—C1	125.17 (17)	C12—C11—H11B	109.2
C4—N1—C1	112.23 (16)	H11A—C11—H11B	107.9
N1—C1—C2B	107.2 (3)	C14—O4—C15	116.71 (18)
N1—C1—C2A	99.7 (2)	C7—C6—C5	119.03 (19)
C2B—C1—C2A	33.3 (3)	C7—C6—C11	119.84 (18)
N1—C1—H1A	111.8	C5—C6—C11	121.13 (18)
C2B—C1—H1A	79.1	O2—N2—O1	122.4 (2)
C2A—C1—H1A	111.8	O2—N2—C8	118.9 (2)
N1—C1—H1B	111.8	O1—N2—C8	118.7 (2)
C2B—C1—H1B	132.2	C13—C12—C14	109.33 (18)
C2A—C1—H1B	111.8	C13—C12—C11	108.76 (17)
H1A—C1—H1B	109.6	C14—C12—C11	110.85 (17)
C3—C2A—C1	105.5 (2)	C13—C12—C4	108.75 (17)
C3—C2A—H2A1	110.6	C14—C12—C4	112.53 (16)
C1—C2A—H2A1	110.6	C11—C12—C4	106.51 (17)
C3—C2A—H2A2	110.6	C10—C9—C8	118.7 (2)
C1—C2A—H2A2	110.6	C10—C9—H9	120.6
H2A1—C2A—H2A2	108.8	C8—C9—H9	120.6
C1—C2B—C3	104.8 (5)	O3—C14—O4	125.2 (2)
C1—C2B—H2B1	110.8	O3—C14—C12	124.4 (2)
C3—C2B—H2B1	110.8	O4—C14—C12	110.42 (17)
C1—C2B—H2B2	110.8	N3—C13—C12	176.2 (3)
C3—C2B—H2B2	110.8	C2A—C3—C4	106.2 (2)
H2B1—C2B—H2B2	108.9	C2A—C3—C2B	33.3 (3)
N1—C5—C10	121.69 (18)	C4—C3—C2B	104.6 (3)
N1—C5—C6	118.85 (18)	C2A—C3—H3A	110.5
C10—C5—C6	119.45 (18)	C4—C3—H3A	110.5
C7—C8—C9	121.76 (19)	C2B—C3—H3A	80.7
C7—C8—N2	118.9 (2)	C2A—C3—H3B	110.5
C9—C8—N2	119.3 (2)	C4—C3—H3B	110.5
C8—C7—C6	120.1 (2)	C2B—C3—H3B	136.8
C8—C7—H7	119.9	H3A—C3—H3B	108.7
C6—C7—H7	119.9	O4—C15—C16	107.2 (2)
C9—C10—C5	120.9 (2)	O4—C15—H15A	110.3
C9—C10—H10	119.5	C16—C15—H15A	110.3
C5—C10—H10	119.5	O4—C15—H15B	110.3
N1—C4—C3	104.31 (17)	C16—C15—H15B	110.3
N1—C4—C12	109.20 (16)	H15A—C15—H15B	108.5
C3—C4—C12	119.69 (19)	C15—C16—H16A	109.5
N1—C4—H4	107.7	C15—C16—H16B	109.5
C3—C4—H4	107.7	H16A—C16—H16B	109.5
C12—C4—H4	107.7	C15—C16—H16C	109.5
C6—C11—C12	112.16 (16)	H16A—C16—H16C	109.5
C6—C11—H11A	109.2	H16B—C16—H16C	109.5
C12—C11—H11A	109.2		
			
C5—N1—C1—C2B	170.8 (4)	C6—C11—C12—C13	167.79 (18)
C4—N1—C1—C2B	−4.9 (4)	C6—C11—C12—C14	−72.0 (2)
C5—N1—C1—C2A	−155.8 (2)	C6—C11—C12—C4	50.7 (2)
C4—N1—C1—C2A	28.5 (3)	N1—C4—C12—C13	−177.03 (18)
N1—C1—C2A—C3	−34.5 (3)	C3—C4—C12—C13	63.0 (3)
C2B—C1—C2A—C3	72.1 (6)	N1—C4—C12—C14	61.7 (2)
N1—C1—C2B—C3	19.3 (6)	C3—C4—C12—C14	−58.3 (3)
C2A—C1—C2B—C3	−62.0 (5)	N1—C4—C12—C11	−60.0 (2)
C4—N1—C5—C10	169.18 (19)	C3—C4—C12—C11	−179.95 (19)
C1—N1—C5—C10	−6.1 (3)	C5—C10—C9—C8	0.1 (3)
C4—N1—C5—C6	−12.2 (3)	C7—C8—C9—C10	−1.4 (3)
C1—N1—C5—C6	172.52 (19)	N2—C8—C9—C10	−179.92 (18)
C9—C8—C7—C6	1.8 (3)	C15—O4—C14—O3	4.6 (3)
N2—C8—C7—C6	−179.62 (19)	C15—O4—C14—C12	−175.16 (19)
N1—C5—C10—C9	179.25 (19)	C13—C12—C14—O3	130.1 (2)
C6—C5—C10—C9	0.6 (3)	C11—C12—C14—O3	10.2 (3)
C5—N1—C4—C3	172.03 (19)	C4—C12—C14—O3	−108.9 (2)
C1—N1—C4—C3	−12.1 (2)	C13—C12—C14—O4	−50.1 (2)
C5—N1—C4—C12	43.0 (3)	C11—C12—C14—O4	−169.98 (16)
C1—N1—C4—C12	−141.17 (18)	C4—C12—C14—O4	70.9 (2)
C8—C7—C6—C5	−1.0 (3)	C14—C12—C13—N3	−151 (4)
C8—C7—C6—C11	179.0 (2)	C11—C12—C13—N3	−29 (4)
N1—C5—C6—C7	−178.85 (19)	C4—C12—C13—N3	86 (4)
C10—C5—C6—C7	−0.2 (3)	C1—C2A—C3—C4	28.8 (3)
N1—C5—C6—C11	1.1 (3)	C1—C2A—C3—C2B	−63.2 (5)
C10—C5—C6—C11	179.78 (19)	N1—C4—C3—C2A	−11.4 (3)
C12—C11—C6—C7	157.10 (19)	C12—C4—C3—C2A	111.1 (3)
C12—C11—C6—C5	−22.9 (3)	N1—C4—C3—C2B	23.2 (4)
C7—C8—N2—O2	−2.7 (3)	C12—C4—C3—C2B	145.6 (4)
C9—C8—N2—O2	175.9 (2)	C1—C2B—C3—C2A	71.0 (6)
C7—C8—N2—O1	177.1 (2)	C1—C2B—C3—C4	−26.4 (6)
C9—C8—N2—O1	−4.3 (3)	C14—O4—C15—C16	172.0 (2)

### Hydrogen-bond geometry (Å, °)

**Table:**

*D*—H···*A*	*D*—H	H···*A*	*D*···*A*	*D*—H···*A*
C11—H11A···O3^i^	0.97	2.48	3.432 (3)	167.

Symmetry codes: (i) −*x*, −*y*+2, −*z*+1.
